# Immediate modulatory effects of transcutaneous vagus nerve stimulation on patients with Parkinson’s disease: a crossover self-controlled fMRI study

**DOI:** 10.3389/fnagi.2024.1444703

**Published:** 2024-10-23

**Authors:** Chengwei Fu, Xiaoyan Hou, Chunye Zheng, Yue Zhang, Zhijie Gao, Zhaoxian Yan, Yongsong Ye, Bo Liu

**Affiliations:** ^1^Department of Acupuncture, Hubei Provincial Hospital of Traditional Chinese Medicine, Wuhan, China; ^2^Department of Acupuncture, Affiliated Hospital of Hubei University of Chinese Medicine, Wuhan, China; ^3^Department of Radiology, The Second Affiliated Hospital of Guangzhou University of Chinese Medicine, Guangzhou, China; ^4^Department of Neurology, The Second Affiliated Hospital of Guangzhou University of Chinese Medicine, Guangzhou, China; ^5^The Second Clinical School, Guangzhou University of Chinese Medicine, Guangzhou, China

**Keywords:** Parkinson’s disease, transcutaneous auricular vagus nerve stimulation, functional magnetic resonance imaging, amplitude of low-frequency fluctuations, neuroimaging, Auricular therapy

## Abstract

**Background:**

Previous studies have evaluated the safety and efficacy of transcutaneous auricular vagus nerve stimulation (taVNS) for the treatment of Parkinson’s disease (PD). However, the mechanism underlying the effect of taVNS on PD remains to be elucidated. This study aimed to investigate the immediate effects of taVNS in PD patients.

**Methods:**

This crossover self-controlled study included 50 PD patients. Each patient underwent three sessions of resting-state functional magnetic resonance imaging (rs-fMRI) under three conditions: real taVNS, sham taVNS, and no taVNS intervention. We analyzed whole-brain amplitude of low-frequency fluctuations (ALFF) from preprocessed fMRI data across different intervention conditions. ALFF values in altered brain regions were correlated with clinical symptoms in PD patients.

**Results:**

Forty-seven participants completed the study and were included in the final analysis. Real taVNS was associated with a widespread decrease in ALFF in the right hemisphere, including the superior parietal lobule, precentral gyrus, postcentral gyrus, middle occipital gyrus, and cuneus (voxel *p* < 0.001, GRF corrected). The ALFF value in the right superior parietal lobule during real taVNS was negatively correlated with the Unified Parkinson’s Disease Rating Scale Part III (*r* = −0.417, *p* = 0.004, Bonferroni corrected).

**Conclusion:**

TaVNS could immediately modulate the functional activity of brain regions involved in superior parietal lobule, precentral gyrus, postcentral gyrus, middle occipital gyrus, and cuneus. These findings offer preliminary insights into the mechanism of taVNS in treating PD and bolster confidence in its long-term therapeutic potential. TaVNS appears to reduce ALFF values in specific brain regions, suggesting a potential modulation mechanism for treating PD.

## 1 Introduction

Parkinson’s disease (PD) is a degenerative disease marked by bradykinesia, tremor, and rigidity, severely impacting patients’ quality of life (GBD 2016 Parkinson’s Disease Collaborators, 2018). As the fastest-growing neurodegenerative disease, global burden of PD has more than doubled in the past 20 years due to increased prevalence, disability, and mortality (GBD 2016 Parkinson’s Disease Collaborators, 2018; Deuschl et al., 2020). Consequently, to mitigate this health burden, efforts to delay disease progression and enhance patient quality of life are crucial.

Current PD treatments include levodopa, dopamine agonists, and monoamine oxidase inhibitors, with levodopa being the primary choice for improving motor function (Jankovic and Tan, 2020; Bloem et al., 2021). However, as the disease progresses, patients often experience motor fluctuations, gait freezing, and levodopa resistance, which complicate treatment and are resistant to conventional therapies (Nonnekes et al., 2016; Hvingelby et al., 2022). Hence, there is a need for novel approaches to reduce movement disorders with fewer side effects.

Neuromodulation techniques, such as transcranial magnetic stimulation (TMS), transcranial direct current stimulation have shown promise as adjuvant therapies for treating PD symptoms (Zhang W. et al., 2022; Broeder et al., 2023). However, these approaches have limitations in targeting deep brain structures affected early in PD pathology (Zhang W. et al., 2022). Vagus nerve stimulation (VNS) is a promising neuromodulation technique used for various neurological and psychiatric disorders, including headaches, depression, and epilepsy (Goggins et al., 2022). Preclinical studies indicate that VNS can enhance locomotor control, reduce neuroinflammation, increase brain-derived neurotrophic factor (BDNF), and mitigate neuronal damage in PD models (Farrand et al., 2017; Farrand et al., 2019; Kin et al., 2021; Hosomoto et al., 2023). Despite its potential, VNS is invasive and requires surgical implantation (Lench et al., 2023). Consequently, noninvasive transcutaneous auricular vagus nerve stimulation (taVNS) has garnered interest (Goggins et al., 2022). Preclinical studies suggest that taVNS can alleviate motor deficits by increasing tyrosine hydroxylase expression and reducing neuroinflammation in 6-OHDA model rats, demonstrating similar efficacy and mechanisms to VNS (Jiang et al., 2018; Kin et al., 2021; Hosomoto et al., 2023). Meanwhile, two clinical studies further supported the safety and efficacy of taVNS in patients with PD (Marano et al., 2022; Lench et al., 2023). These findings highlight taVNS as a promising neuromodulator for PD treatment, with growing interest in its mechanisms.

Resting-state functional magnetic resonance imaging (rs-fMRI) is a technique developed for studying the intrinsic neurological activity of individuals. The amplitude of low-frequency fluctuation (ALFF) is a rs-fMRI analysis method that reflects the intensity of spontaneous neural activity in brain regions (Zang et al., 2007). Our recent research suggests that ALFF is crucial for understanding taVNS efficacy. We found that taVNS modulates the sensorimotor network in insomnia patients and the default mode network in migraine patients (Wu et al., 2021; Fu et al., 2022). Additionally, taVNS affects the cognitive control network and salience network in depression (Sun et al., 2023). Based on these findings, we hypothesize that ALFF may also reflect the mechanisms of taVNS in PD. This study aims to explore the immediate effects of taVNS in PD patients using a crossover design with three fMRI scanning sessions (baseline, real taVNS, and sham taVNS). We will compare ALFF differences among conditions to test the hypothesis that taVNS modulates brain function in PD patients. This research may provide insights into the neural mechanism of taVNS for treating PD.

## 2 Methods

### 2.1 Participants

This single-blinded, randomized, self-controlled study included 50 participants diagnosed with Parkinson’s disease (PD) between April 2021 and October 2022. Participants were recruited from the Outpatient Department of Parkinson Disease at the Second Affiliated Hospital of Guangzhou University of Chinese Medicine. The study protocol was registered on the Chinese Clinical Trial Registry.^1^ Informed consent was obtained from all participants.

### 2.2 Eligibility criteria

Inclusion Criteria

1. Diagnosis of PD according to the UK Brain Bank Parkinson’s Disease criteria (Wei et al., 2022).
2. Stable-dose anti-PD treatment for at least 2 months before enrollment.
3. No history of antidepressant treatment (e.g., psychotherapy, medication therapy, electroconvulsive shock).
4. No use of analgesics, anesthetics, or hypnotics for at least 1 month before the screening visit.
5. Right handed.
6. Medication OFF state during clinical measurement and fMRI scanning.

Exclusion Criteria

1. Other forms of parkinsonism or secondary PD.
2. History of major psychiatric illnesses (e.g., schizophrenia, somatization disorder) or antipsychotic drugs (e.g., olanzapine, risperidone).
3. History of dementia or severe cognitive dysfunction according to the Mini-Mental State Examination (MMSE) score (MMSE ≤24) (Li et al., 2016).
4. MRI evidence of cerebrovascular disease, epilepsy, tumor, or other organic brain lesions.
5. I Internal mental implants (e.g., insulin pump, cardiac pacemaker) or history of brain operation (e.g., Gamma Knife, deep brain stimulation).
6. Patients with a history of other clinically significant medical illnesses or drug abuse.
7. Allergies to metal or damage/inflammation at the auricle.
8. Pregnant or lactating;
9. Participation in other clinical research trials within the last 6 months.

### 2.3 Clinical assessment

All participants were assessed using the Movement Disorder Society Unified Parkinson’s Disease Rating Scale (UPDRS), which includes 4 parts reflecting nonmotor experiences of daily living (UPDRS-I), motor experiences of daily living (UPDRS-II), motor examination (UPDRS-III) and motor complications (UPDRS-IV). Motor dysfunction scores were calculated from UPDRS-III for both left and right sides.

Quality of life and non-motor symptoms were evaluated using the 39-item Parkinson’s Disease Questionnaire (PDQ), the 30-item Nonmotor Symptoms Scale (NMSS), and the 12-item Mini-Mental State Examination (MMSE), respectively.

### 2.4 Intervention and randomization

**Figure 1:**
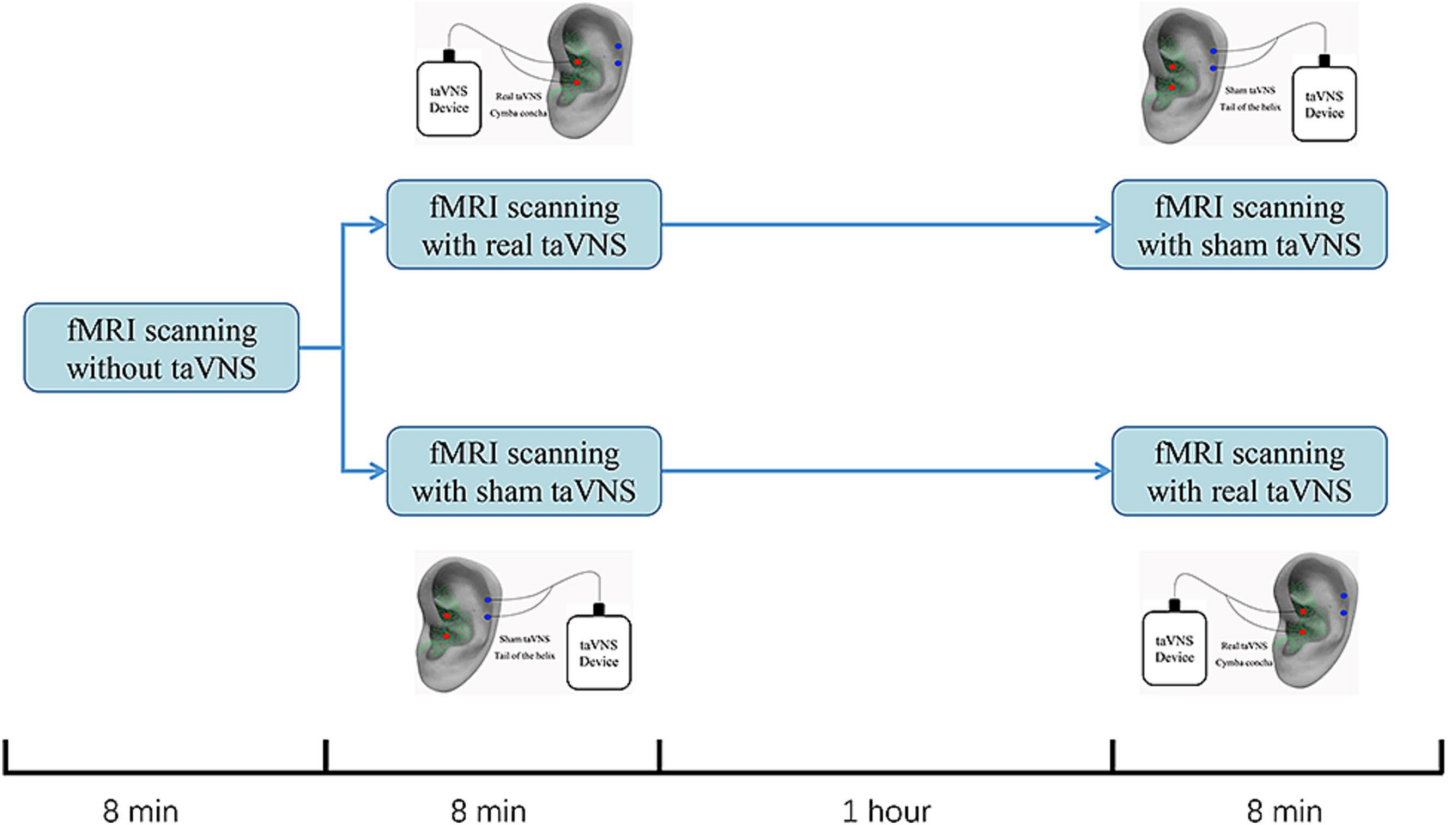
Study design for sham-controlled transcutaneous auricular vagus nerve stimulation (taVNS) within the fMRI scanning. All participants completed the study on one testing days. Participants would first receive an 8-min baseline fMRI scanning without any stimulation. Then, they were randomized into 2 group. One would receive an 8-min fMRI scanning with real taVNS followed by an 8-min fMRI scanning with sham taVNS. The other group would change the order of the real and sham taVNS. The interval between real and sham taVNS is 1 h.

An electric device (Hwato, SDZ-IIB, Suzhou, China) with an enhanced stimulator, used in previous studies, was employed for the intervention (Figure 1; Wu et al., 2021; Zhang et al., 2021). Real taVNS was applied at the left cymba concha, while sham taVNS was applied at the left tail of the helix. Both interventions used the same stimulation parameters: a dilatational wave (on–off cycle stimulation) of 20/100 Hz with a pulse width of 0.2 ms. Intensity was adjusted based on participants’ subjective feelings (usually 1 mA to 2.5 mA) (Fu et al., 2022; Wen et al., 2023). To minimize the effect of the intervention sequence, a 1:1 crossover protocol was used (Shen et al., 2020; Borgmann et al., 2021). Participants will first undergo an 8-min baseline fMRI scan without any stimulation. They will then be randomized into two groups: one group will receive 8 min of real taVNS followed by 8 min of sham taVNS, with a 1-h interval between the two scans. The other group will receive 8 min of sham taVNS followed by 8 min of real taVNS, also with a 1-h interval between the scans All treatments were conducted by an experienced acupuncturist who did not participate in final statistical analysis.

### 2.5 MRI data acquisition

All participants underwent three fMRI scans on a 3.0-T Philips Ingenia MR scanner with a standard 32-channel head coil. Before scanning, patients wore earplugs to reduce the noise of the scanner. Earplugs were used to reduce scanner noise, and sponge pads were fixed around the head to minimize motion. Participants were instructed to stay quiet, awake, and with eyes closed. After scanning, experienced neuroradiologists verified routine T2 MRI for abnormalities.

A three-dimensional, high-resolution T1-weighted sequence was used to acquire whole-brain structural data, whose parameters were as follows: acquisition time = 314 s matrix = 320 × 300 × 280 slices, repetition time (TR) = 1.0 ms, echo time (TE) = 4.7 ms, slice gap = 0 mm, flip angle = 8°, field of view (FOV) = 256 × 240 × 224, voxel size = 0.8 × 0.8 × 0.8 mm. Functional images were obtained with the following parameters: acquisition time = 486 s matrix = 64 × 61 × 38 slices, TR = 2000 ms, TE = 30 ms, slice gap = 0.25 mm, dynamic scans = 240, flip angle = 90°, FOV = 240 × 240 × 142, voxel size = 3.75 × 3.75 × 3.5 mm. The parameters of T2-weighted were as follows: TR = 748 ms, TE = 30 ms, FOV = 230 mm × 183 mm, matrix = 256 × 163, flip angle = 8°, slice thickness = 60 mm, slice = 25, acquisition time = 123 s.

### 2.6 Data preprocessing

Functional images were preprocessed using CONN 21.0 (Whitfield-Gabrieli and Nieto-Castanon, 2012). For each participant’s data, the first 10 volumes of each condition were discarded, followed by slice timing correction using the middle slice as a reference. Realignment was performed to correct for rigid body-motion. Individual EPI images were coregistered to individual T1 images and spatial normalization to the standard Montreal Neurological Institute template with a resample resolution of 2 × 2 × 2-mm voxels. Data were smoothed with a Gaussian kernel at a full width at half maximum of 6 mm. Subjects with mean frame displacement >0.2 mm were excluded to reduce motion effects, as in our previous study (Zhang et al., 2021). We identified outlier time points in the motion parameters and global signal intensity using ART implemented with the CompCor procedure. Linear regression model was used to regress out the confounding factors (head motion parameters of the 24-parameter model, white matter, cerebrospinal fluid and outliers) from the time series of each voxel, and the linear trends were removed.

### 2.7 ALFF analysis

ALFF analysis was performed using the CONN-fMRI toolbox. After the aforementioned preprocessing, a bandpass filter (0.01–0.08 Hz) was applied to extract the time series of each voxel and convert them to the frequency domain using fast Fourier transform. The ALFF value is defined as the square root of the power spectrum. Finally, a Z-score transformation was used for the ALFF for convenient subsequent statistical analysis.

### 2.8 Statistical analysis

Repeated measures ANOVA was used to evaluate differences in the ALFF among the conditions. The statistical significance thresholds were defined as *p* < 0.001 (voxel level, uncorrected) and *p* < 0.05 (cluster level, Gaussian random field (GRF) corrected). Because the voxel values in the statistic image are not spatially independent due to inherent properties of the MR imaging process, functional connections in brain and BOLD signal properties, the family-wise error rate correction would give conservative thresholds. While GRF correction gives fewer conservative tests for estimating and taking into account the spatial smoothness which is a widely used multiple comparisons correction method in fMRI studies (Tillikainen et al., 2006; Fasiello et al., 2022). Then, we extracted the mean ALFF value of each cluster in each condition post-hoc in SPSS 24.0. Only the clusters whose ALFF value in the real taVNS condition were significantly different from those in both the sham taVNS condition and the baseline condition were analyzed for correlations between the ALFF values and clinical scale scores using Pearson correlation. A threshold of *p* < 0.05 (Bonferroni corrected) was applied for multiple comparisons in the correlation analysis.

## 3 Results

### 3.1 Demographic and clinical data

Out of 50 participants recruited, all completed the fMRI scan and clinical assessments. Quality control excluded 3 participants due to head motion with frame displacement (FD) > 0.2 mm. Thus, 47 PD patients were included in the analysis. Tables 1, 2 present the demographic and clinical data of these participants. The lateralization of motor symptoms was not significant. Most PD patients were free of motor complications. None of them had dementia or cognitive impairment.

**Table 1:** Demographic of included PD patients (*n* = 47).

Age (year), mean ± sd	64.04 ± 9.06
Sex (male/female), n	20/27
Height (cm), mean ± sd	160.72 ± 8.29
Weight (kg), mean ± sd	57.27 ± 9.52
Duration of PD (month), mean ± sd	70.23 ± 40.82
Education	
Primary education, n	6
Junior middle education, n	9
Senior middle education, n	16
Undergraduate, n	15
Postgraduate, n	1

**Table 2:** Clinical assessments of included PD patients (*n* = 47).

UPDRS-total, mean ± sd	57.55 ± 22.48
UPDRS-I, mean ± sd	9.11 ± 4.04
UPDRS-II, mean ± sd	10.26 ± 5.20
UPDRS-III, mean ± sd	35.74 ± 15.01
UPDRS-III right site, mean ± sd	12.23 ± 5.73
UPDRS-III left site, mean ± sd	11.81 ± 6.71
UPDRS-IV, median (IQR)	0 (0, 4)
Hoehn-Yahr, median (IQR)	2 (1.5, 2.5)
NMSS, mean ± sd	32.28 ± 19.38
PDQ, mean ± sd	22.77 ± 11.96
MMSE, median (IQR)	29 (27, 30)

UPDRS, Unified Parkinson’s Disease Rating Scale; NMSS, Nonmotor Symptoms Scale; PDQ, Parkinson’s Disease Questionnaire; MMSE, Mini-Mental State Examination.

### 3.2 Effects of taVNS on ALFF in PD patients

**Figure 2:**
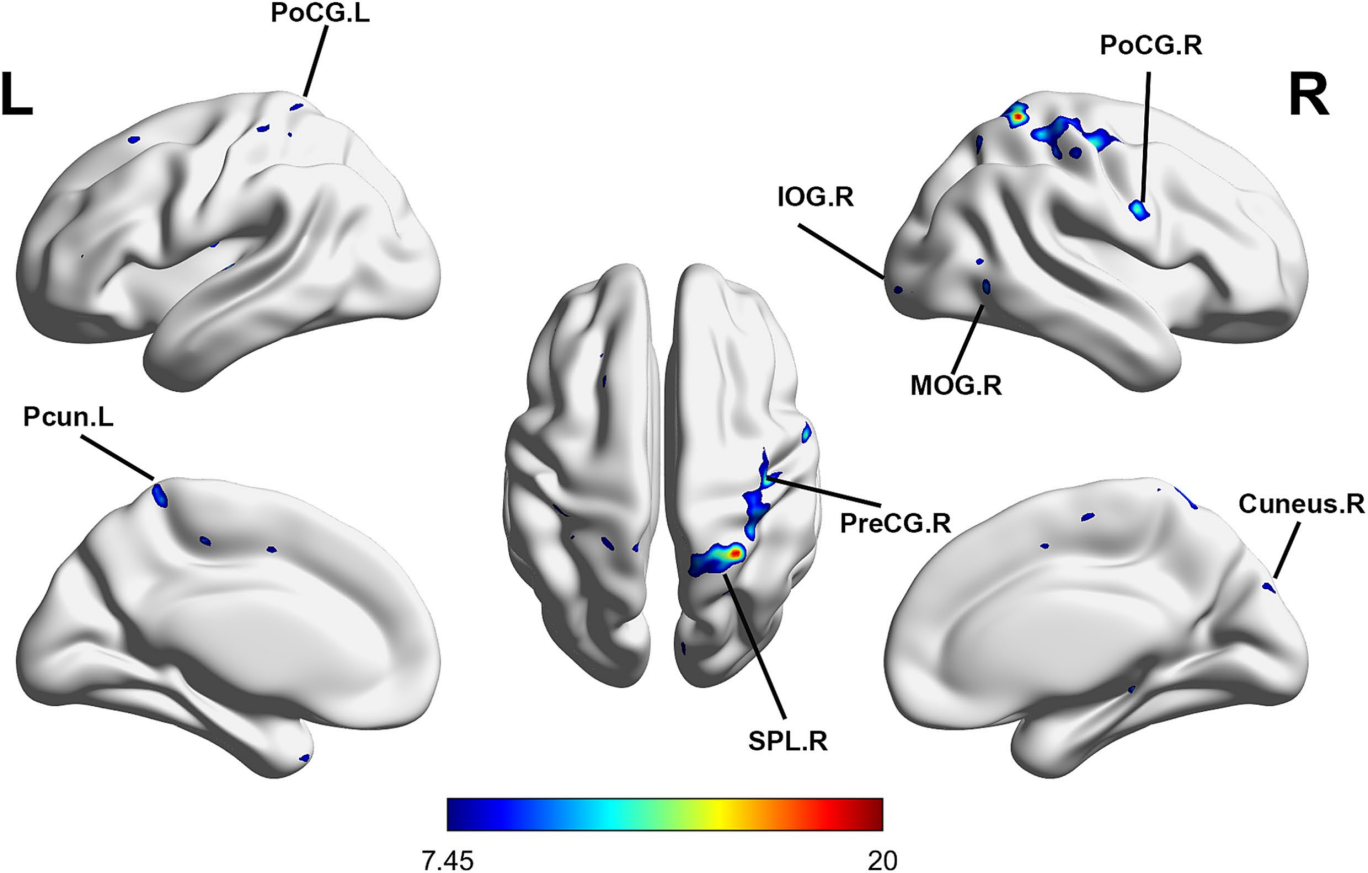
Significant ALFF changes among baseline, sham taVNS, and real taVNS conditions (voxel *p* < 0.001, cluster *p* < 0.05, GRF corrected). Significant ALFF changes were observed in the right Inferior Occipital Gyrus (IOG), right Middle Occipital Gyrus (MOR), right Precentral Gyrus (PreCG), right Cuneus, right Postcentral Gyrus (PoCG), right Superior Parietal Lobule (SPL), left PoCG, and left Precuneus (PCUN).

**Figure 3:**
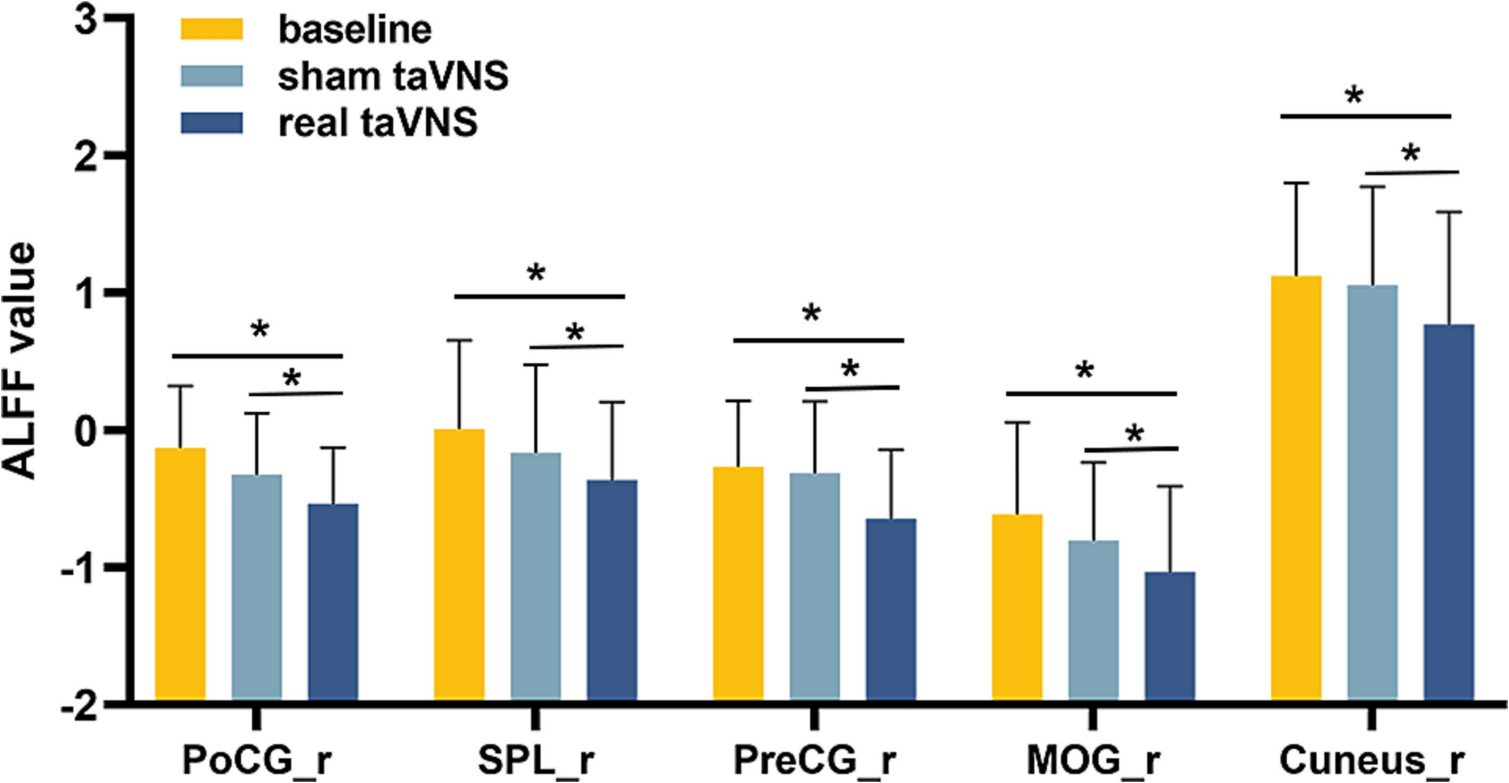
ALFF values in PD patients under different conditions (means ± SD). Compared to baseline and sham taVNS conditions, a significant decrease in ALFF was found in the real taVNS condition in the right Middle Occipital Gyrus (MOR), right Precentral Gyrus (PreCG), right Postcentral Gyrus (PoCG), right Superior Parietal Lobule (SPL), and right Cuneus. * means significant difference (*P* < 0.05) between two groups.

Repeated-measures ANOVA identified 8 clusters with significant differences among conditions. These clusters included the right inferior occipital gyrus (IOG), right middle occipital gyrus (MOR), right precentral gyrus (PreCG), right cuneus, right postcentral gyrus (PoCG), right superior parietal lobule (SPL), left PoCG, and left precuneus (PCUN) (Table 3). Figure 2 illustrates cluster locations. Post-hoc analysis of ALFF values revealed that the right MOR, right PreCG, right PoCG, right SPL, and right cuneus showed significantly lower ALFF values in the real taVNS condition compared to baseline and sham taVNS conditions (Figure 3 and Supplementary File 1).

**Table 3:** Significant clusters among different conditions on ALFF in PD patients.

Cluster	Brain regions	MNI peak			Voxel size	Peak intensity
		X	Y	Z		
Cluster 1	IOG_r	34	−98	−4	109	18.6119
Cluster 2	MOG_r	36	−90	14	38	11.2884
Cluster 3	PreCG_r	58	−2	28	46	14.6726
Cluster 4	Cuneus_r	8	−86	40	37	11.2434
Cluster 5	PoCG_r	26	−28	74	662	15.9707
Cluster 6	SPL_r	28	−48	64	248	20.2738
Cluster 7	PoCG_l	−42	−36	64	41	10.2287
Cluster 8	Pcun_l	−10	−44	66	39	12.366

IOG_r, right inferior occipital gyrus; MOG_r, right middle occipital gyrus; PreCG_r, right precentral gyrus; PoCG_r, right postcentral gyrus; SPL_r, right superior parietal lobule; PoCG_l, left postcentral gyrus; Pcun_l, left precuneus.

### 3.3 Correlation analysis

**Figure 4:**
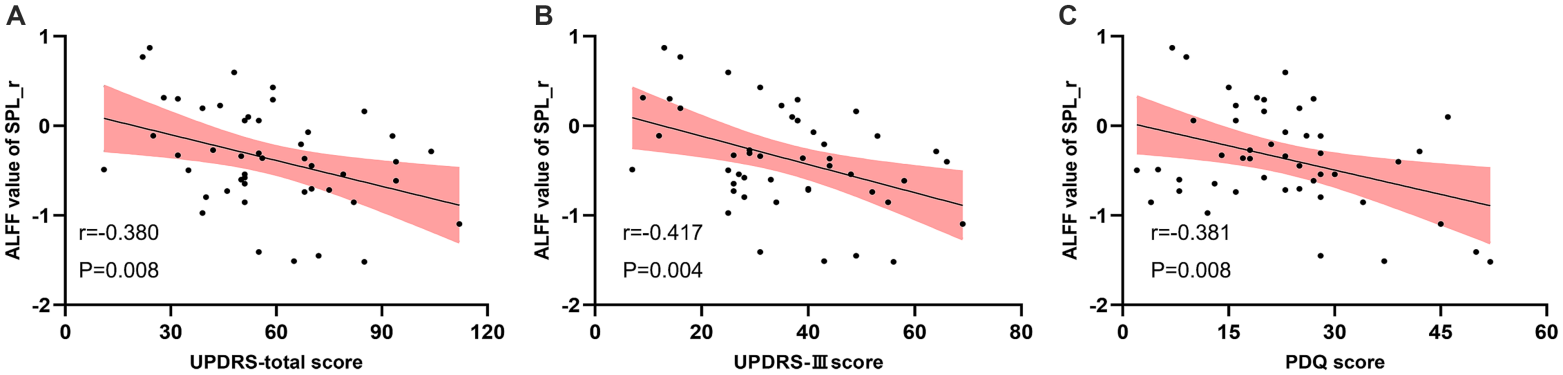
Negative correlation between the ALFF value of the real taVNS condition in the right Superior Parietal Lobule (SPL) and clinical assessments. **(A)** ALFF value in right SPL negatively correlates with UPDRS score (*r* = −0.380, *p* = 0.008). **(B)** ALFF value in right SPL negatively correlates with UPDRS-III score (*r* = −0.417, *p* = 0.004). **(C)** ALFF value in right SPL negatively correlates with PDQ score (*r* = −0.381, *p* = 0.008).

Pearson correlation analysis indicated that the ALFF of right SPL in the real taVNS condition was negatively correlated with the total UPDRS score (*r* = −0.380, *p* = 0.008, Bonferroni corrected), UPDRS-III score (*r* = −0.417, *p* = 0.004, Bonferroni corrected), and PDQ score (*r* = −0.381, *p* = 0.008, Bonferroni corrected) (Figure 4).

## 4 Discussion

This study assessed the immediate impact of taVNS on PD using ALFF. Our results demonstrated that taVNS reduced ALFF across several brain regions, including the PreCG, PoCG, SPL, MOG, and cuneus. Notably, the ALFF value in the right SPL during real taVNS was negatively correlated with the UPDRS total score, UPDRS-III score, and PDQ score, suggesting that taVNS may influence PD by modulating widespread brain activity.

A recent study revealed that, compared with healthy controls, patients with PD showed increased ALFF in the PreCG and PoCG (Yang et al., 2023). The first finding of our research is that the abnormal ALFF changes in the PreCG and PoCG in PD patients can be decreased by taVNS. PreCG and PoCG belong to the sensorimotor network (SMN), which integrates sensory inputs to assist motor program execution (Chi et al., 2023). Neuroimaging studies have repeatedly shown disease-related alterations in the SMN in patients with PD. Tessitore and colleagues applied different analytic approaches and found that alterations within the SMN were consistently reported across PD stages (Strafella et al., 2018). Alterations in the SMN are well-documented in PD, with evidence indicating that disruptions in this network are associated with impaired sensory integration and motor function (Pan et al., 2017; Caspers et al., 2021). These disruptions are considered key aspects of PD pathophysiology and treatment response (Strafella et al., 2018). SMN regulation can be a partial target of PD treatment. Recent research also highlights those interventions targeting the SMN, such as TMS, can improve motor symptoms by affecting network centrality (Chi et al., 2023). Moreover, levodopa and pramipexole, as the primary prescriptions of PD medication, may also be associated with the regulation of SMN (Esposito et al., 2013; Ye et al., 2017). Our study supports the potential of taVNS as a therapeutic approach for PD by modulating SMN activity, suggesting that taVNS might be an effective treatment strategy to address SMN dysfunction in PD.

Another important finding in our study was that real taVNS application downregulated the ALFF of SPL in patients with PD, suggesting that taVNS may have modulatory effects on brain function. SPL covers the dorsal part of the posterior parietal cortex, anatomically connecting the parietal lobe and occipital lobes, composed of occipital cytoarchitectural pattern and parietal cytoarchitectural pattern (Caspers et al., 2012; Sulpizio et al., 2023). The special cellular components and topographic position determined SPL play a key role in sensorimotor tasks, particularly in visuomotor tasks. Some brain imaging studies have shown that the function of SPL in shifting spatial attention between locations, perceiving and planning travel paths during locomotion, and tracking motion under attentional load (Alahmadi, 2021; Biagi et al., 2022). Meanwhile, some studies investigate SPL was activated earlier than PreCG and induces motor status, which further confirms the role of SPL in movement control (Zhang Q. et al., 2022; Zhang et al., 2023). Several studies have revealed SPL is involved in the pathological process of movement impairment in PD patients. One study compared the potentials of SPL during a series of motor tasks in patients with PD and showed that the beta power of SPL was specifically inhibited from standing to walking and negatively correlated with walking speed (Zhang et al., 2023). Another study investigated the increased functional connectivity between cerebellum and SPL would in future development of freezing of gait in PD patients (Jung et al., 2020). Gu’s meta-analysis included 10 studies that compared whole-brain ALFF between PD patients and healthy control (Gu et al., 2022). Thus, regulating the function of SPL may be useful for PD treatment. Therefore, modulating SPL function could be beneficial for PD treatment. Previous research has demonstrated that transcranial direct current stimulation (tDCS) over 12 months can enhance regional cerebral blood flow in the SPL, which is reduced in PD patients compared to healthy controls (Song et al., 2020; Wu et al., 2024). Our finding that real taVNS reduced ALFF in the SPL and its negative correlation with the UPDRS-III score, but not with baseline ALFF, suggests that SPL’s response to taVNS might be more pronounced under specific conditions. This indicates that SPL may affect motor function in particular states or stages of PD, warranting further investigation through task-based fMRI studies.

What’s more, the occipital cortex, central to visual perception and processing, plays a role in PD. Impaired visual networks in PD patients have been linked to more severe motor deficits (Lan et al., 2023). PD patients with freezing gait showed impairments in the occipital lobule (Tessitore et al., 2012). To better control movement, PD patients rely more on visual cues (Uc et al., 2005; Wang et al., 2023). Previous studies have found that abnormal ALFF changes in the occipital cortex are common in PD patients. Li et al. reported that the decrease in CBF/ALFF in the bilateral middle occipital gyrus along the dorsal visual stream in PD patients was negatively correlated with motor impairments, which was due to increased ALFF in MOG (Li et al., 2023). Lan et al. (2023) observed increased ALFF value of left MOG in PD patients was positively correlated with postural instability/gait difficulty symptoms. Furthermore, Hu et al. (2015) reported that as PD worsened, the fractional ALFF of MOG continually increased. Based on the above studies, a reasonable assumption is that hyperactivation of the occipital cortex may be due to compensatory mechanisms for impaired motor function in PD patients (Lan et al., 2023). Correspondingly, a potential target of PD treatment is to lower the ALFF of the occipital cortex. Xiong’s study revealed that after performing MRI-guided focused ultrasound, motor impairment reduction was associated with a decrease in fractional ALFF values of the left occipital cortex (Xiong et al., 2022). In this study, we found that the real taVNS decreased the ALFF of MOG and cuneus, while sham taVNS did not, supports this hypothesis. Moreover, other studies have suggested that the effect of taVNS is associated with modulating the function of the occipital cortex (Zhao et al., 2020). Thus, taVNS may improve PD symptoms by downregulating spontaneous activity in the occipital cortex.

Interestingly, the majority of our study’s findings were localized to the right hemisphere. Initially, we considered that this might relate to the asymmetrical nature of PD, which often presents with unilateral onset and suggests uneven damage between the two hemispheres. Because PD clinically manifests as unilateral onset, indicating asymmetrical damage between the two hemispheres (Yuan et al., 2022). Some studies have investigated the lateralization of PD using voxel-mirrored homotopic connectivity (VMHC) and found a widespread decrease in VMHC in PD patients (Zhu et al., 2016; Liao et al., 2020; Goggins et al., 2022; Yuan et al., 2022; Zhang H. et al., 2022). However, our study found no significant difference in symptoms between the left and right sides in the PD patients we included. This suggests that the lateralization observed might be more related to the effects of taVNS rather than to symptom lateralization. Previous research has indicated that taVNS may have lateralized effects. For example, Capone et al. (2015) demonstrated increased cortical excitability in the right motor cortex following real taVNS applied to the left cymba concha. Similarly, Chen et al. (2022) found that reaction times in left-difficult tasks improved and EEG changes were observed after taVNS applied to the left cymba concha. If the lateralization effect of taVNS is consistently demonstrated, it could limit the applicability of taVNS if used only on the left side. However, since we did not investigate the effects of right-sided taVNS in our study, there is currently no direct evidence linking the lateralization effect specifically to taVNS.

Several limitations should be noted. First, our study was a self-controlled design without a healthy control group, which means changes observed in the brain may not be exclusive to PD patients. Future studies with healthy controls are needed to validate our findings. Second, our analysis focused solely on the off-medication state. While clinical assessments are often performed on-medication, off-medication evaluations and fMRI scans are more common in clinical trials to minimize the effects of dopamine therapy on brain activation (Harrington et al., 2017; Ragothaman et al., 2023). Finally, our study only examined the short-term effects of taVNS. Long-term effects and more detailed mechanisms require further investigation.

## 5 Conclusion

This study demonstrated that immediate taVNS reduces resting-state brain activity in the right hemisphere, affecting regions such as the PreCG, PoCG, SPL, MOG, and cuneus in PD patients. These preliminary results suggest a potential mechanism for taVNS in PD treatment. Future research should investigate the long-term effects of taVNS and explore how these changes in brain activity correlate with clinical outcomes in PD patients.

## Data Availability

The raw data supporting the conclusions of this article will be made available by the authors, without undue reservation.

## Footnotes

^1^ https://www.chictr.org.cn/showproj.html?proj=56715
