# Corrigendum: Immediate modulatory effects of transcutaneous vagus nerve stimulation on patients with Parkinson's disease: a crossover self-controlled fMRI study

**DOI:** 10.3389/fnagi.2024.1538891

**Published:** 2024-12-17

**Authors:** Chengwei Fu, Xiaoyan Hou, Chunye Zheng, Yue Zhang, Zhijie Gao, Zhaoxian Yan, Yongsong Ye, Bo Liu

**Affiliations:** ^1^Department of Acupuncture, Hubei Provincial Hospital of Traditional Chinese Medicine, Wuhan, China; ^2^Department of Acupuncture, Affiliated Hospital of Hubei University of Chinese Medicine, Wuhan, China; ^3^Department of Radiology, The Second Affiliated Hospital of Guangzhou University of Chinese Medicine, Guangzhou, China; ^4^Department of Neurology, The Second Affiliated Hospital of Guangzhou University of Chinese Medicine, Guangzhou, China; ^5^The Second Clinical School, Guangzhou University of Chinese Medicine, Guangzhou, China

**Keywords:** Parkinson's disease, transcutaneous auricular vagus nerve stimulation, functional magnetic resonance imaging, amplitude of low-frequency fluctuations, neuroimaging, Auricular therapy

In the published article, there was an error in the affiliation numbers of authors “Xiaoyan Hou, Yue Zhang, Zhijie Gao, Zhaoxian Yan, Yongsong Ye, and Bo Liu.” Instead of being affiliated with “Department of Acupuncture, Hubei Provincial Hospital of Traditional Chinese Medicine, Wuhan, China,” these authors should be affiliated with “Department of Radiology, The Second Affiliated Hospital of Guangzhou University of Chinese Medicine, Guangzhou, China.”

The authors apologize for this error and state that this does not change the scientific conclusions of the article in any way. The original article has been updated.

