# Corrigendum: Immediate modulatory effects of transcutaneous vagus nerve stimulation on patients with Parkinson's disease: a crossover self-controlled fMRI study

**DOI:** 10.3389/fnagi.2025.1560826

**Published:** 2025-01-30

**Authors:** Chengwei Fu, Xiaoyan Hou, Chunye Zheng, Yue Zhang, Zhijie Gao, Zhaoxian Yan, Yongsong Ye, Bo Liu

**Affiliations:** ^1^Department of Acupuncture, Hubei Provincial Hospital of Traditional Chinese Medicine, Wuhan, China; ^2^Department of Acupuncture, Affiliated Hospital of Hubei University of Chinese Medicine, Wuhan, China; ^3^Department of Radiology, The Second Affiliated Hospital of Guangzhou University of Chinese Medicine, Guangzhou, China; ^4^Department of Neurology, The Second Affiliated Hospital of Guangzhou University of Chinese Medicine, Guangzhou, China; ^5^The Second Clinical School, Guangzhou University of Chinese Medicine, Guangzhou, China

**Keywords:** Parkinson's disease, transcutaneous auricular vagus nerve stimulation, functional magnetic resonance imaging, amplitude of low-frequency fluctuations, neuroimaging, auricular therapy

In the published article, there was an error in the author list. Chengwei Fu, Xaoyan Hou and Chunye Zheng made equal contributions to this work, and this should have been indicated with the symbol “†” but this was erroneously omitted. The corrected author list appears below:

Chengwei Fu^1, 2, 3†^, Xiaoyan Hou^3†^, Chunye Zheng^4†^, Yue Zhang^3^, Zhijie Gao^3, 5^, Zhaoxian Yan^3^, Yongsong Ye^3^ and Bo Liu^3*^

^†^These authors have contributed equally to this work

The authors apologize for this error and state that this does not change the scientific conclusions of the article in any way. The original article has been updated.

