# Supplementary material for: Immediate modulatory effects of transcutaneous vagus nerve stimulation on patients with Parkinson’s disease: a crossover self-controlled fMRI study
Source: Front Aging Neurosci. 2024 Oct 23;16:1444703. doi: 10.3389/fnagi.2024.1444703 (PMC11537911; doi:10.3389/fnagi.2024.1444703)
Supplement: Supplementary file 1 [file Table_1.docx]

| Table S1 ALFF value of each cluster in different stimulation condition | | | | | | |
| --- | --- | --- | --- | --- | --- | --- |
|  | Baseline | Sham taVNS | Real taVNS | F | P value | η2 |
| PoCG_r | -0.13±0.45 | -0.33±0.45 | -0.54±0.41 | 25.531 | ＜0.001 | 0.357 |
| IOG_r | -0.06±0.63 | -0.45±0.64 | -0.53±0.68 | 14.796 | ＜0.001 | 0.243 |
| SPL_r | -0.01±0.65 | -0.17±0.64 | -0.36±0.57 | 14.769 | ＜0.001 | 0.243 |
| PreCG_r | -0.27±0.48 | -0.31±0.52 | -0.65±0.50 | 18.203 | ＜0.001 | 0.284 |
| PoCG_l | 0.38±0.79 | 0.03±0.84 | 0.76±0.77 | 11.469 | ＜0.001 | 0.200 |
| Pcun | -0.41±0.42 | -0.61±0.58 | -0.80±0.49 | 14.232 | ＜0.001 | 0.236 |
| MOG_r | -0.62±0.67 | -0.81±0.57 | -1.03±0.62 | 12.727 | ＜0.001 | 0.217 |
| Cuneus_r | 1.12±0.68 | 1.05±0.72 | 0.77±0.82 | 12.089 | ＜0.001 | 0.208 |
|  | | | | | | |
